# Impact and Assessment of Research Integrity Teaching: A Systematic Literature Review

**DOI:** 10.1007/s11948-024-00493-1

**Published:** 2024-07-23

**Authors:** Daniel Crean, Bert Gordijn, Alan J. Kearns

**Affiliations:** 1 School of Veterinary Medicine, University College Dublin, Belfield, Dublin 4, Ireland; 2 Conway Institute of Biomolecular and Biomedical Science, University College Dublin, Dublin, Ireland; 3 Institute of Ethics, School of Theology, Philosophy, and Music, Dublin City University, Dublin, Ireland

**Keywords:** Research integrity, Teaching, Education, Assessment, Ethical decision-making

## Abstract

Presented here is a systematic literature review of what the academic literature asserts about: (1) the stages of the ethical decision-making process (i.e. awareness, reasoning, motivation, and action) that are claimed to be improved or not improved by RI teaching and whether these claims are supported by evidence; (2) the measurements used to determine the effectiveness of RI teaching; and (3) the stage/s of the ethical decision-making process that are difficult to assess. Regarding (1), awareness was the stage most claimed to be amenable to improvement following RI teaching, and with motivation being the stage that is rarely addressed in the academic literature. While few, some sources claimed RI teaching cannot improve specific stages. With behaviour (action) being the stage referenced most, albeit in only 9% of the total sources, for not being amenable to improvement following RI teaching. Finally, most claims were supported by empirical evidence. Regarding (2), measures most frequently used are custom in-house surveys and some validated measures. Additionally, there is much debate in the literature regarding the adequacy of current assessment measures in RI teaching, and even their absence. Such debate warrants caution when we are considering the empirical evidence supplied to support that RI teaching does or does not improve a specific stage of the decision-making process. Regarding (3), only behaviour was discussed as being difficult to assess, if not impossible. In our discussion section we contextualise these results, and following this we derive some recommendations for relevant stakeholders in RI teaching.

**Supplementary Information:**

The online version contains supplementary material available at 10.1007/s11948-024-00493-1.

## Introduction

Scientific research is an integral part of society, influencing not only how society functions today, but how it can improve in the future, both socially and technologically (Sørensen et al., 2021). Trust in the integrity of science is essential for the continued positive contribution of science to society (Caelleigh, 2003; D'Souza et al., 2020; Sørensen et al., 2021). Since the mid-1980s interest has increased in the area of Research Integrity (RI) (Kennedy et al., 2018). This has been matched by intensive research spanning from what exactly RI is, to what ethical and pedagogical approaches should be used in teaching it to foster a culture of integrity (Kalichman & Plemmons, 2007; Kennedy et al., 2018; Mejlgaard et al., 2020; Resnik & Elliott, 2019; Sørensen et al., 2021; Steneck, 2006; Turrens, 2005; Valkenburg et al., 2021; Zahari et al., 2021). Concomitantly, reports have confirmed numerous problematic research practices, which include, but are not limited to, salami-slicing, cherry picking of data, sloppy research, and a culture of ‘publish or perish’ (Combating Scientific Misconduct, 2011; Ellis, 2015; Gopalakrishna et al., 2022; Kennedy et al., 2018; O'Hara, 2011; Steneck, 2002; Wheeler, 1989). Such practices are leading to problems such as erroneous data and misinformation, and if unresolved may negatively affect society’s trust in science (Altman, 1994; Baker, 2016; Brainard, 2018; Freedman, 2010; Prinz et al., 2011). 

RI is encouraged through several ways including statutory regulations, professional bodies and their codes of practice, incentives, sanctions, and numerous education and training programs (Crean et al., 2023; Kabasenche, 2014; Kennedy et al., 2018; Labib et al., 2021; Mejlgaard et al., 2020; Pizzolato & Dierickx, 2021). The aspirations of these education and training programs, which we term as RI teaching hereafter, span from RI teaching sensitising researchers to RI issues, to the promotion and fostering of ethical behaviour (Antes & DuBois, 2014). Rest (1986) distinguishes four stages of the ethical decision-making process, i.e. awareness, reasoning, motivation, and action. While not specifically in reference to Rest (1986), these stages are often mentioned as aims / goals of RI teaching (Dubois & Dueker, 2009; Goddiksen & Gjerris, 2022; Labib et al., 2022, 2023). However, which of these four stages RI teaching is claimed to be able to improve or not, and whether these claims are supported by evidence, is not always apparent. Moreover, while many RI courses exist, evidence for how effective these courses are, and how we assess effectiveness itself, is limited (Abdi et al., 2021; Katsarov et al., 2022; Marušić et al., 2016). Here, we have set out to understand how these important areas are discussed in the academic literature, identify where further attention is warranted, and derive some recommendations from the findings for relevant stakeholders in RI teaching. To that end, this systematic review aims to identify what the academic literature asserts about:

1. the stages of the ethical decision-making process (i.e. awareness, reasoning, motivation, and action) that are claimed to be improved or not improved by RI teaching and whether these claims are supported by evidence;
2. the measurements used to determine the effectiveness of RI teaching; and
3. the stage/s of the ethical decision-making process that are difficult to assess.

## Methodology

This review was guided by the Preferred Reporting Items for Systematic Reviews and Meta-Analyses (PRISMA) method (http://www.prisma-statement.org) (Sarkis-Onofre et al., 2021). For this review, the multidisciplinary databases Scopus and Web of Science (WoS) core collection were used to find relevant sources (search updated as of September 2023). Scopus and WoS are believed to be two of the most comprehensive and widely used bibliographic databases in the scientific community (Pranckutė, 2021; Zhu & Liu, 2020). As such we, the authors, concluded that the use of these two comprehensive multidisciplinary databases along with a backward snowballing-based approach outlined below was sufficient for the purpose of this study. No limitations on document type or year were applied.

**Fig. 1:**
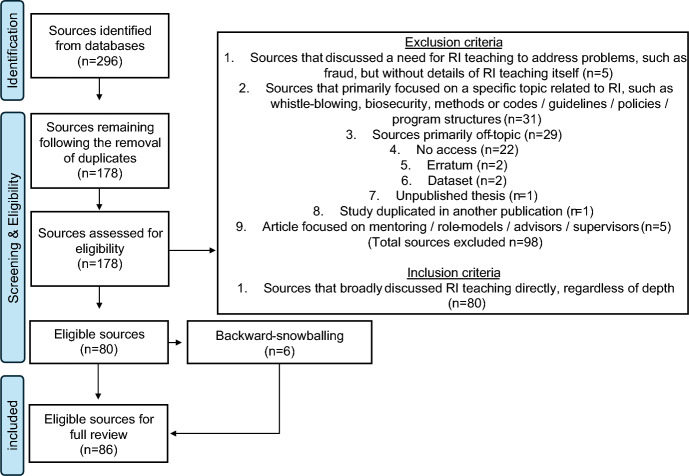
PRISMA guided flowchart of the search criteria used to identify eligible sources

Figure 1 below shows a flowchart of the search strategy used to identify relevant sources. Sources containing the words ‘research integrity’ OR ‘scientific integrity’ OR ‘responsible conduct of research’ AND ‘training’ OR ‘educat*’ OR ‘teach*’ in their title were initially selected. Wildcards (highlighted using an asterisk ‘*’) were used to capture variations of terms, for example education, educating, or teaching and so on. By definition, education and training can differ on many levels, for example the methods or duration, as discussed very well by Garavan (1997). However, for our purpose, we were interested in the examination of our aims independent of these different factors, and therefore our search included education, training, and teaching. The rationale being that we were not focused on what approaches or type of teaching is effective or more effective than another—other authors have asked and answered this question (Antes et al., 2010; Katsarov et al., 2022; Marušić et al., 2016; Watts et al., 2017). We were primarily focused on how RI teaching in its entirety affects any stage of the ethical decision-making process (i.e. awareness, reasoning, motivation, and action). 140 and 156 sources were retrieved from Scopus and WoS, respectively. Following the removal of duplicates a total of 178 sources remained (Supplementary File (SF) 1, Page 2-sources). Following an initial screening of each source, exclusion and inclusion criteria were then applied, as shown in Fig. 1, resulting in 80 sources deemed eligible for full review (see SF1, page 3-ineligible sources for details of all 98 excluded sources). Additionally, a backward snowballing-based approach produced a further six eligible sources (SF1, Page 4-backward-snowballing) (see SF1, page 5-eligible sources for details of all 86 eligible sources). The lead author conducted all screening and reviewing of sources independently, and discussed each stage with the entire author team for agreement. At every stage all authors discussed and agreed on all aspects of the study, including: the aims, search methodology, screening and exclusion / inclusion criteria, results, discussion, conclusion and recommendations.

**Fig. 2:**
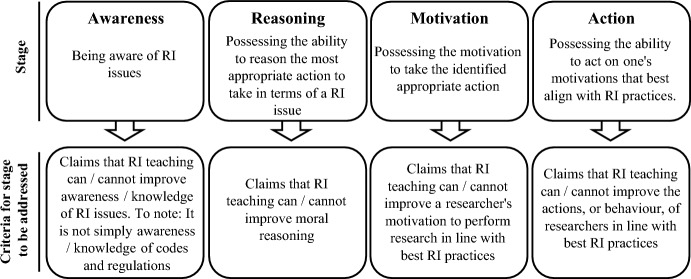
Rubric for identifying whether sources discussed how RI teaching addressed the four stages within an ethical decision-making process. This rubric was guided by the stages as defined by Rest (1986)

As shown in Fig. 2 below, a rubric was established to address aim 1: the stages of the ethical decision-making process (i.e. awareness, reasoning, motivation, and action) that are claimed to be improved or not improved by RI teaching and whether these claims are supported by evidence. Following a reading of all eligible sources, it was observed that ‘behaviour’ was primarily the term used for action. For examples of sources using the term ‘behaviour’, please see SF1, page 6-stages column AD which contains further details. We also examined whether sources provided evidence in support for claims. Evidence was deemed as either primary (i.e. the source itself contains quantitative / qualitative data) or secondary (i.e. the source references a primary source or a review that directly evaluates primary sources, e.g. a meta-analysis). For this review, we did not include details of how mentors, role-models, advisors, or supervisors have an impact on the four stages. Rather we focused on formal RI teaching.

Regarding aim 2: the measurements used to determine the effectiveness of RI teaching, we have identified five means of assessing the effectiveness of RI teaching contained within the eligible sources. These were unvalidated surveys, validated surveys / questionnaires, portfolio / assignment-based, interviews, and observational methods. To note, regarding validated measures, we did not examine whether the measures used were appropriate relative to the course content, but only whether the test itself was a known validated measure.

## Results

### Aim 1: The Stage(s) of the Ethical Decision-Making Process (i.e. Awareness, Reasoning, Motivation, and Action) that are Claimed to be Improved or Not Improved by RI Teaching and Whether These Claims are Supported by Evidence

44 sources contained claims for improvement or lack of improvement toward one or more stages of the decision-making process following RI teaching (see SF1, page 6 column E and corresponding references in column D). 36, 11, 6, and 13 sources claimed that RI teaching improves awareness, reasoning, motivation, and action respectively (see SF1, page 6 columns F, G, H, and I and corresponding references in column D). The claims for improved awareness, reasoning, motivation, and action were supported by evidence in 31 sources (86% of total claims), 11 sources (100% of total claims), 4 sources (66% of total claims), and 13 sources (100% of total claims), respectively (see SF1, page 6 columns J, K, L, and M and corresponding references in column D). Please see the methods section of this paper for details of what was deemed as evidence (i.e. primary or secondary). Also, please see SF1, page 6 columns N, O, P, Q, Z, AA, AB, and AC, and corresponding references in column D, for the identification of the sources with evidence, and the type of evidence (i.e. either primary or secondary).

2, 4, 3, and 8 sources claimed that RI teaching cannot improve awareness, reasoning, motivation, and action, respectively (see SF1, page 6 columns R, S, T and U and corresponding references in column D). The claims that RI teaching cannot improve awareness, reasoning, motivation, and action were supported by evidence in 2 sources (100% of total claims), 4 sources (100% of total claims), 1 source (33% of total claims), and 8 sources (100% of total claims), respectively (see SF1, page 6 columns V, W, X and Y and corresponding references in column D).

### Aim 2: The Measurements Used to Determine the Effectiveness of RI Teaching

Assessing the effectiveness of RI teaching is mentioned in 63 sources, to varying degrees (see SF1, page 7 column E and corresponding references in column D). 29 of these 63 sources (46%) maintained that assessment strategies are absent and need to be identified or are in place but are inadequate (see SF1, page 7 column F and corresponding references in column D). These 29 sources are evenly spread across all years captured in our search. Discussions relating to the inadequacy / absence of measures in general were contained within 23 sources, 7 sources had specific points in relation to surveys, and 2 in relation to interviews (see SF1, page 7 column G and H and corresponding references in column D). Details of these discussions are shown in SF1, page 7 column H and are summarised in Table 1 (SF1, page 8-Table 1). Several sources also acknowledged the need for assessment measures, but without further elaborations on the subject (Dubois & Dueker, 2009; Goddiksen & Gjerris, 2022; Koterwas et al., 2021; Plemmons & Kalichman, 2013; Sponholz, 2000; Tessier, 2019; Wester, 2007).

Unvalidated surveys, validated surveys / questionnaire-based, interviews, portfolio / assignment-based, and observational analysis were mentioned as part of an assessment approach for the effectiveness of RI teaching in 31, 12, 5, 7 and 3 sources respectively (See SF1 page 7 columns J, K, L, M and N and corresponding references in column D). 38 sources contained measures that were developed in-house (which includes all unvalidated surveys), and 12 contained validated measures (see SF1 page 7 columns P and Q and corresponding references in column D). The validated measures identified were: DIT, DIT2, EDM, ESIT, field-specific DIT, MJS, MJT, Path2Integrity Questionnaire, PCM, PCS, PDR, PDV, PKS, PMT, PSS, RCRM, REKASA, SMARTS, SRRS, TESS (see SF1 page 7 column O for the full name of the abbreviated validated tests and corresponding reference in column D).

### Aim 3: The Stage/s of the Ethical Decision-Making Process that are Difficult to Assess

We next examined whether any source specified that a stage of the decision-making process was difficult to assess. 13 sources in total claimed action (within all sources described as behaviour) was difficult to assess (see SF1, page 7, column I and corresponding references in column D). 8 sources did not give a detailed rationale but simply acknowledged that changes in behaviour (action) are difficult to assess (Brown & Kalichman, 1998; Chua et al., 2022; Diaz-Martinez et al., 2021; Evans et al., 2023; Hooper et al., 2018; Pizzolato & Dierickx, 2021; Plemmons et al., 2006; Van den Hoven et al., 2023). 5 sources gave a rationale as to why behaviour is difficult to assess, and these are summarised in Table 2 (SF1, page 9-Table 2).

## Discussion

*Improvability of the ethical decision-making process* All stages of the ethical decision-making process were claimed to be addressed by RI teaching, with more claims of improvement than no improvement across the stages. Sources addressed how RI teaching improves awareness mostly, followed by action (behaviour), reasoning and motivation (36, 13, 11 and 6 sources, respectively). It is clear within our sources that the distribution of the stages being addressed by RI teaching is not uniform. Interestingly, motivation is the stage with the least claims for improvement, and with only 3 claims for no improvement. However, as discussed in the PRINTEGER project’s normative analysis document (Meriste et al., 2016), motivation, and not simply satisfying commitments due to external incentives, is critical to acting with integrity. Moreover, external incentives will invariably change from time to time, and a reliance on them as a driver for RI may not promote a stable RI environment, which is driven from within the research community.

Several authors have questioned whether RI teaching can improve the latter two stages at all, i.e. motivation and action (Plemmons & Kalichman, 2013; Sarauw, 2021; Steneck, 2013). Furthermore, our results here show that claims that RI teaching can / cannot improve action / behaviour are conflicting, and from this it might be inferred that improvements in behaviour are relative to the course content, which are known to vary greatly (Fanelli, 2019; Marušić et al., 2016). Or, perhaps this conflict arises from the fact that most studies use surrogate markers of behaviour (action) by using self-reporting. However, as we will discuss later, this self-reporting may confound the reliability of the evidence. It is reasonable to conclude that RI teaching can improve the first two stages (i.e. awareness and reasoning), while motivation and action are more difficult, given that they are confounded by multiple factors outside of the control of specific RI teaching, for example the overall research culture (Labib. et al., 2022; Resnik, 2014). Labib et al. (2022) further expands our understanding of motivation in terms of motivating individuals to firstly engage with RI education, and not simply motivation to do the right thing. While here we have focused on motivation to do the right thing following RI teaching, we should not underappreciate the role for motivating individuals to want to engage with RI teaching in the first place, which could undoubtedly impact on its effectiveness. While we have identified that specific stages are being addressed, and evidence is being supplied to support claims for improvement (or lack of) following RI teaching, the credibility of this evidence will hinge on the appropriateness of the assessment measures being used. This bring us to our second aim in this study where we have identified the assessment measures that are available.

*Assessment measures* Our results demonstrate that the assessment measures being used are predominantly in-house surveys followed by some validated survey / questionnaire-based measures. However, there is much discussion in our sources surrounding the adequacy, and at times absence, of appropriate assessment measures. Interestingly, Sachs and Siegler (1993), and Van den Hoven et al. (2023), one of the earliest and most recent publications in our sources, are both included in sources addressing issues with assessment measures, illustrating that the issue has been discussed for some time and is still ongoing. As shown in SF1, page 8-Table 1 these discussions spanned several areas such as the logistical difficulties in assessing; that the lack of clear aims, goals or learning outcomes and diversity of RI teaching all hinder the development of appropriate assessment measures, and that there is a lack of evidence-based guidance on how to evaluate (for further details and references see SF1, page 8-Table 1).

Surveys were primarily the assessment measure where weaknesses were directly discussed, and it appears to the authors here that these weaknesses are valid, albeit surveys are still frequently used. These weaknesses included a level of ambiguity around what the answers are *actually* measuring, possible participant self-selection bias, and a recognition that socially desirable responding may influence the trustworthiness of the responses given to questions. Additionally, how people perceive themselves may not always reflect how they *actually* behave. This is evidenced in a study by Sarauw et al. (2021), where survey results of participants showed that 93% *believed* they were honest and trustworthy, while 73% of the same participants admitted that they *had* recently lied or cheated. Authors also discussed both the benefits and the weaknesses of interviews, noting that they allowed greater insight into the thinking of the course participants compared to surveys for example, but interviews may be subject to self-selection bias, to small sample sizes, are time consuming and require a certain skill to perform and interpret effectively (McGee et al., 2008; Seiler et al., 2011).

*Validation and appropriateness* Several validated assessment measures also exist and were identified within our sources, primarily in the form of surveys / questionnaires. However, while no direct weaknesses in these measures themselves were discussed, it is unknown whether these validated measures are actually appropriate measures relative to the course content. For example, as Antes and DuBois (2014) question, how can a course that focuses on knowledge and rules be assessed appropriately using a measure such as the Defining Issues Test (DIT). While these validated measures are available, sources within our study claim that the evidence available on the effectiveness of measures is limited (Antes et al., 2010; Hooper et al., 2018; Todd et al., 2017). There is also an assertion in our sources that behaviour is difficult to assess, if not impossible, given that the behaviour changes sought from RI teaching take place in real world scenarios outside of specific RI teaching courses, impacted by numerous external factors, such as mentors, as well as the technical and financial costs associated with collecting the behavioural data itself (See SF1, page 9-Table 2). While behavioural change is ultimately the intended end goal of RCR, when we consider the difficulty in assessing behaviour, perhaps efforts should concentrate on what can be achieved, as noted by Labib et al. (2022), in terms of subjective process evaluations. We also note that how motivation is assessed is not directly discussed in our sources. Considering the importance of motivation, as mentioned earlier in this discussion referencing the PRINTEGER project’s normative analysis, this appears to be an area that does need to be given more consideration.

*The need for prudence* As already mentioned, not only is there a diverse array of courses (Fanelli, 2019; Marušić et al., 2016), external factors such as mentoring can also impact on course outcome, rendering the establishment of a standardised assessment measure more difficult. However, whether such standardisation is possible or necessary is not clear when we consider in-house developed assessments may at times be more appropriate (Diaz-Martinez et al., 2019). Diaz-Matinez et al. (2019) claim that while validated measures are encouraged they may not be the best option for all teaching, some of which may require in-house developed measures to appropriately assess their specific learning objectives. If assessing RI teaching requires specific in-house developed measures, which requires a particular skill set and time, we may have to concede that such measures are most likely not transferable or scalable. Yet, considering the global nature of research today it could be more efficient and desirable to focus on measures that are in some way transferable and scalable. And with that, measures that can be applied in institutes where funding and / or time are constrained. However, this would require a more global alignment for the intended goals of RI teaching, which at present is lacking (Steneck, 2013). Perhaps the use of the taxonomy for research integrity training (TRIT) described by Van den Hoven et al. (2023) would be helpful in guiding course design, including goals, and the selection of appropriate assessment measures. Reflecting on these discussions, prudence should be applied when considering which assessment measure/s to use and the evidence supplied to claim RI teaching can / cannot improve, for example, a specific stage of the ethical decision-making process. The assessment measure should not just be a validated test, but a test that is valid for assessing the content of the course in question. If this is not the case, it will be difficult to know whether courses are successful or not, or simply not assessed appropriately to reach any meaningful conclusions. Moreover, there are several other variables which can impact on course outcome, for example, the ethics approach used (e.g. duty-based, consequentialist-based, or virtue-based), the target audience (e.g. undergraduate, postgraduate, or principal investigators), and the delivery medium (e.g. online, face-to-face or hybrid), to name but a few. It is imperative to further examine the best approaches to positively impact specific stages of the ethical decision-making process, for example when considering a specific audience or via specific course content or delivery methods. And in order to achieve this we must be able to identify appropriate assessment measures.

## Conclusion and Recommendations

Within this study we have identified that the four stages of the ethical decision-making process are claimed to be amenable to improvement by RI teaching despite a small number of studies claiming they are not. However, the four stages are not addressed in a uniform manner. Claims regarding the improvement of awareness due to RI teaching are addressed in 36 out of the total 86 sources (42%). However, the other stages are not addressed as well. Motivation is the least addressed stage, as it was mentioned in only 6 out of the total 86 sources (7%). Each stage of the ethical decision-making process is important. And while it may be more feasible to assess awareness for example, and perhaps this is a reason for the lack of uniformity, the other stages should be addressed more. We have identified that most claims regarding the effectiveness of RI teaching for a given stage of the ethical decision-making process are supported by some form of empirical evidence. However, we have also identified in this study that there is a lack of certainty on whether the assessment measures used are always appropriate, which can shed doubt on such evidence. Upon reflection of these observations, we recommend that further attention is warranted in relation to the assessment measures themselves and derive the following recommendations:

1. At present, there is no clear criteria for how an assessment measure is judged to be validated for RI teaching. For authors and those involved directly in developing RI teaching approaches, it is essential to be able to identify the criteria being used for a measure to be termed validated for RI teaching. This knowledge deficit should be addressed and examination of what possible assessment measures are appropriate for RI teaching must be identified and their validity examined.
2. Following on from recommendation 1, authors and those involved directly in developing RI teaching should determine what validated assessment measures are the most appropriate to use for a specific outcome (e.g. a specific stage of the ethical decision-making process).
3. We have identified that how RI teaching impacts some stages of the ethical decision-making process, for example motivation and action (behaviour), are not addressed adequately. Some may believe that certain stages are outside of the achievable learning outcomes following RI teaching, such as behaviour in a real-world scenario. And while this may be true, it is not clear to the authors here that there is sufficient empirical evidence to allow any stage, at present, to be neglected. We recommend that all stages of the ethical decision-making process are adequately addressed in terms of how they are impacted by RI teaching. We are mindful that for this recommendation to be achieved, recommendation 1 and 2 must first come to fruition, and we must know how to assess effectiveness appropriately.

### Limitations

First limitation: We have focused primarily on how formal RI teaching improves, or does not improve, a specific stage of the ethical decision-making process. From a reading of the sources, it was clear that some stages were claimed to be influenced by informal RI teaching, such as mentors influencing the behaviour of mentees in particular. With the rationales for such influence being that mentees in many cases are exposed to a mentor’s behaviour in terms of RI for prolonged periods of time, reinforcing good or bad practice and ethical / unethical behaviour (Bell, 2015; Pizzolato & Dierickx, 2021; Wester et al., 2010). If we had considered informal RI teaching, we would have observed a small increase in claims regarding stages affected by RI teaching, particularly behaviour (action).

Second limitation: We have also included studies which made claims regarding how RI teaching affected behaviour. Most of these studies used surrogate markers of behaviour, e.g. self-perception of what one might do in the future, which sources acknowledged are indeed not measurements of *actual* behaviour. Considering that assessing actual behaviour changes is difficult – as discussed previously – we have included studies using surrogate markers as they may be the most feasible option for measuring potential behaviour in a RI teaching setting and are worth identifying.

Third limitation: It is unclear what criteria are being used to justify an assessment measure being termed as a validated measure specifically for RI teaching. While we have termed certain measures validated, either by them being claimed as validated in the source or are commonly known to be validated, for example the DIT, it is not clear that specific studies have addressed what qualifies as a validated measure specifically for Responsible Conduct of Research (RCR).

Fourth limitation: Although we have identified that certain claims are supported by empirical evidence, we have not dissected the actual evidence supplied to determine its validity.

Fifth limitation: While we have identified that behaviour (action) is the only stage that is discussed as being difficult to assess, this does not mean that the other stages are not difficult, but simply that they have not been discussed with respect to assessment difficulty.

Sixth limitation: Having more than one screening author can be useful in the systematic review search method, to reduce certain limitation such as studies being missed (Waffenschmidt et al., 2019). However, in this review only one author, albeit with consistent communication between all authors at all stages, performed the screening. While we are confident that we have captured an appropriate volume of relevant sources we acknowledge this as a possible limitation.

### Supplementary Information

Below is the link to the electronic supplementary material.

Supplementary file1 (XLSX 274 KB)
